# Clinical gait analysis and physical examination don’t correlate with physical activity of children with cerebral palsy. Cross-sectional study

**DOI:** 10.1080/23335432.2020.1812429

**Published:** 2020-09-03

**Authors:** Anne-Laure Guinet, Neijib Khouri, Eric Desailly

**Affiliations:** a Informatics, Bioinformatics, Complex Systems Lab, University of Paris-Saclay, Gif-sur-Yvette, France; b Pôle Recherche et Innovation, Fondation Ellen Poidatz, Ellen Poidatz Research Lab, Saint Fargeau-Ponthierry, France; c Chirurgie orthopédique pédiatrique, Centre Hospitalier Universitaire Necker-Enfants Malades Hospital, Paris, France

**Keywords:** Cerebral palsy, gait analysis, physical activity, daily activity, biomechanics

## Abstract

Gait analysis and physical clinical measures are usually performed in children with cerebral palsy to help the surgeons make therapeutic decision. However, the level of physical activity in daily life is not systematically assessed. The aim of this cross sectional study was to examine the correlations between: three-dimensional gait analysis kinematic and spatiotemporal parameters, clinical measures and physical activity. Participants were 30 children with cerebral palsy (10–18 y), with GMFCS I–III. Daily physical activity was measured with an Actigraph GT3X accelerometer in free living environment during seven consecutive days. The percent of time spent in sedentary, in moderate to vigorous physical activity and the number of steps per day were computed from the accelerometer data. Kinematics parameters did not correlate with physical activity. Moderate correlations were found between spatio-temporal parameters and physical activity, for instance timing of toe-off (r = −0.40, p = 0.03). Few physical examination parameters were correlated with physical activity, such as the hip flexors selective motor control (r = 0.69 with moderate to vigorous activity and r = 0.70 with steps per day, p < 0.05). The physical activity profile cannot be sufficiently determined by a combination of clinical measures.

## Introduction

Cerebral palsy (CP), which is the largest cause of motor disability in childhood (Sellier et al. 2016), describes a group of permanent disorders of the development of movement and posture, causing activity limitation, that are attributed to non-progressive disturbances to the developing foetal or infant brain (Rosenbaum et al. 2007). Motor disorders of CP are often accompanied by secondary musculoskeletal problems, such as reduced joint range of motion, increased joint stiffness and muscle weakness. These secondary alterations progress with age and contribute to a gradual loss of functional capacity, characterised by deterioration in gait and reduced muscular strength throughout adolescence and young adulthood in individuals with CP (Bleasdale 1984; Johnson et al. 1997).

In the context of planning treatments (include single-event multi-level surgery), a specific evaluation of walking is performed preoperatively using a three-dimensional gait analysis, to assist in surgical decision-making (Gage 1983). Gait analysis provides information on the kinematic, kinetic and spatio-temporal parameters of walking. A multivariate measure of overall gait pathology, such as Gait Deviation Index (GDI), a kinematic based index of overall gait pathology, have been developed to facilitate the interpretation of quantitative results (Schwartz and Rozumalski 2008). In parallel with this instrumented examination, the clinician performs a neuro-orthopaedic assessment (i.e. clinical measures) measuring passive joint range of motion, structural deformations, muscular strength, spasticity and selective motor control. Multiple parameters are evaluated by these two exams.

In addition to the motor disorders, it appears that children with CP practice less physical activity than typically developing children (TD) (Bjornson et al. 2007; Maher et al. 2007; Zwier et al. 2010; Ryan et al. 2015). PA is defined by any bodily movement produced by skeletal muscle that results in energy expenditure (Caspersen et al. 1985). Lack of physical activity may contribute to the development of chronic pain, obesity, fatigue and osteoporosis. For children and teenagers, physical activity includes play, sports, travel, daily chores, recreational activities, physical education or planned exercise, in the family, school or community (World Health Organization 2010). Participation in exercise and in physical activity, for children with CP, contributes to maintain health and fitness, and also it is essential to physical development. (Fowler et al. 2007) The level of daily physical activity may be recorded with an accelerometer that has been validated as an objective measure of ambulatory physical activity in children and adolescents with CP (Capio et al. 2010; Clanchy et al. 2011; Gorter et al. 2012). The parameters commonly recorded are the total step count per day and the percentage of time spent in moderate to vigorous physical activity (MVPA) and in sedentary (SED). This registration is made daily, for 7 days (Ishikawa et al. 2013).

If one of the aims of the treatments is to improve activity and participation, including physical activity level, their specific assessments are not systematically done. One of the reasons for this lack of evaluation could be that assessing physical activity is time consuming. Our hypothesis is that clinicians assume that gait analysis and clinical examination are sufficiently relevant and are correlated to the physical activity level of their patient. They could also think that a patient who walk better may have higher PA and conversely.

But, the question of the relationship between the physical activity of children with CP and the clinical examination and the gait analysis remains open. Evaluating specifically physical activity level in children with CP is therefore critical. Its interest is twofold: firstly, the objective assessment of PA provides better information about global child’s health (impairments of body, activity limitations and participation restriction) and could improve the clinical diagnosis; second, it could help clinicians to propose better treatments and to optimally care for these children and youth.

Consequently, the aim of this cross sectional study was to examine the correlations between: three-dimensional gait analysis, clinical measures and physical activity and to evaluate the combined correlated value of clinical measures on the physical activity profile of children with CP.

Recently, Wilson et al. (Wilson et al. 2015) showed that there is a correlation between GDI and moderate physical activity (r = 0.47) and a high physical activity (r = 0.51). They also demonstrated a high correlation between GDI and the average steps per day (r = 0.58). In their study, Wilson et al. did not assess the correlations with a set of kinematic parameters nor with clinical examination data.

Considering the multi-dimensional nature of physical activity, it is likely that physical activity does not depend solely on the intrinsic parameters of the subject. As a result, the hypotheses of the study are that there are no correlations between the parameters of the gait analysis, the clinical assessment and physical activity, and that there are no relationships between physical activity, gait analysis and clinical assessment variables.

## Method

### Participants

**Figure 1.**
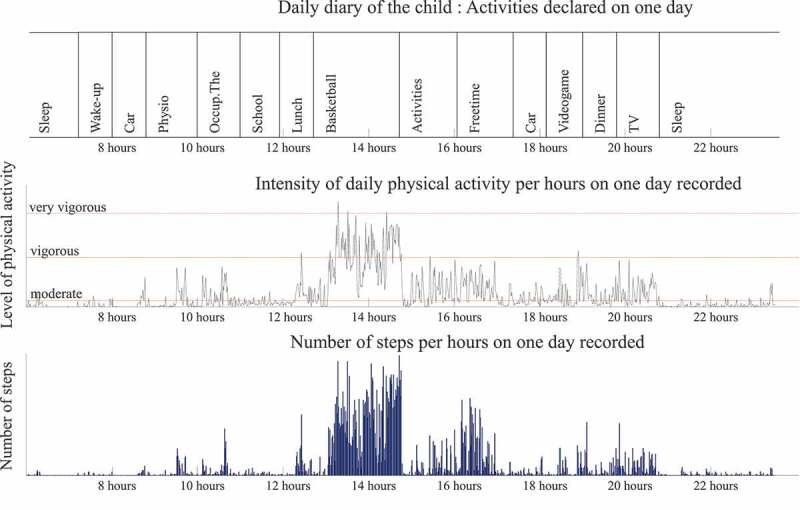
Flow diagram presents detailed information on the process of recruiting study participants

Inclusion criteria were: ambulatory and community living children with CP, aged 10–18 years, who functioned at Gross Motor Function Classification System I–III, and who were undergoing a clinically indicated three-dimensional gait analysis for future treatment (including single event multi-level surgery or botulinum neurotoxin treatment). Children were not included if they had significant illnesses, injury or surgery within the last 18 months which may have impacted on usual activity levels in the community, and if they were unable to complete gait analysis because of cognitive deficit or behaviour disorder. Detailed information on the process of recruiting study participants is exposed in the flow diagram (Figure 1). Written consent was obtained from each child’s parent or guardian and assent from each child. The study was approved by the French independent ethic committee (N°19071004), and by the French National Agency regulating Data Protection (N°1997047V0). Data collection took place between January to August 2016.

### Procedure

For this exploratory cross-sectional study, each participant wore the accelerometer Actigraph GT3X® in free-living situation to record daily physical activity. They were also examined by three-dimensional gait analysis followed by standardized clinical examination in the Foundation Ellen Poidatz gait lab. All explicative variables are summarized in Appendix 1.

#### Accelerometer

**Figure 2.**
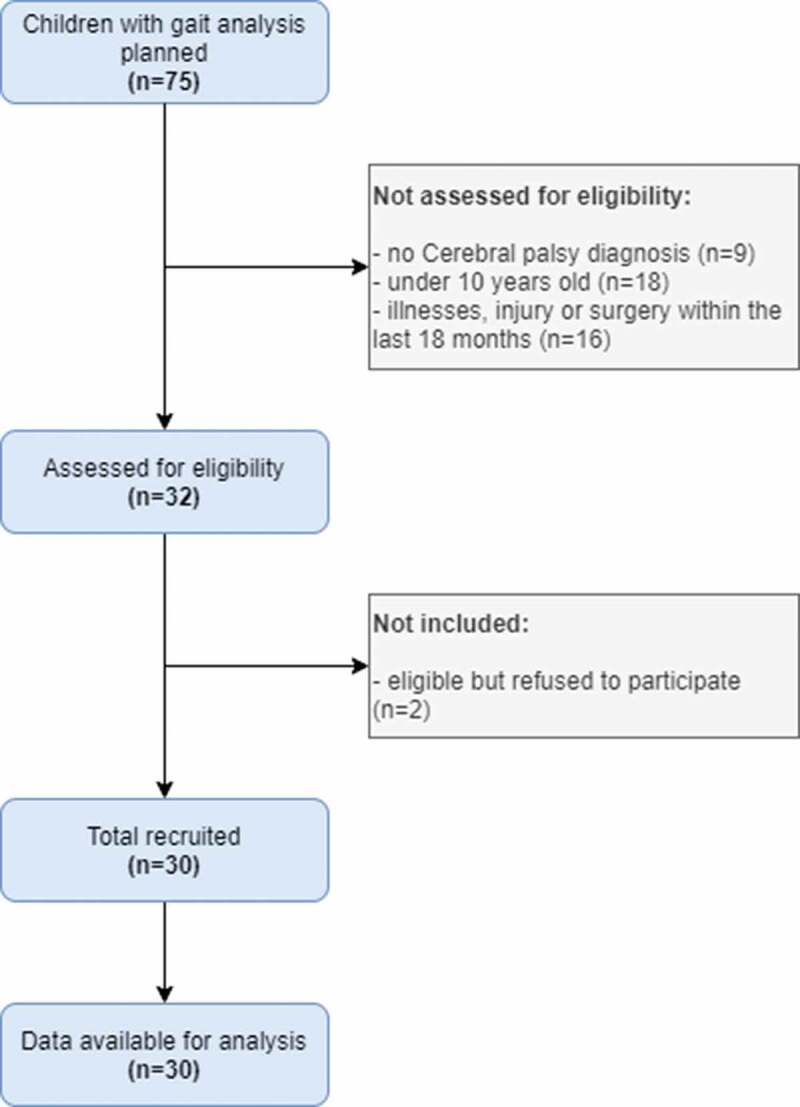
Example of daily diary, steps and intensity of physical activity (PA) for one child on one day. A previous verification was made between daily diary and data recorded by accelerometer to validate the wear time. We noticed high level of PA during break time and sport activities, paradoxically moderate to vigorous activity is recorded during videogame, dinner and TV at home. The daily diary was completed by the patient, and it does not discriminate the activities (maybe the child played with active videogame, and they did not specify). Globally, this child spent 18% of his week in MVPA and he made an average of 7187 steps per day

The Actigraph® captures and records human activity information using a solid state three-axis accelerometer. Acceleration data from all participants were collected with a 100-Hz sampling frequency. The output recorded by the Actigraph® (counts per minutes) is converted into units of PA intensity using specific algorithm (Freedson et al. 2005). The parameters used in this study were percentage spent in Sedentary, in moderate to vigorous physical activity, and step per day. ‘MVPA’ is used when referring to the amount of time a patient spends above a ‘Moderate’ cut point level, thus indicating significant activity, and it is a category of activity intensity that has been consistently shown to reduce the risk of many chronic disease states (Hajna et al. 2017). A Freedson’s children equation was used to translate and interpret the accelerometer signal, in Actilife® software. Freedson cut-off point is acceptable to estimate the time spent in MVPA and in SED (Freedson et al. 2005; Trost et al. 2006). Children wore the accelerometer around the waist for seven days (Ishikawa et al. 2013), holidays excluded for all children. Recording was not made simultaneous between children. During the week recorded by the accelerometer, parents or children had to complete a daily diary. They described hour by hour, all activities and sports they practiced. A wear-time validation was made using Actilife software, by applying an automatic filter which remove all period with zero acceleration signals. After, a manual systematic screening of the data was completed between data recorded by the accelerometers and the activities reported in the diary in order to optimize the wear-time validation (Figure 2). For example, if the child wrote that he or she spent the day at an amusement park, the day was manually deleted as this could skew the results.

#### Gait analysis

Kinematic measurements were collected using a fifteen-camera VICON system (8 MX 20, 5 T 40, 2 T 160) (PluginGait marker set, VICON, Oxford Metrics, UK), and four KISTLER force plates (Kistler Holding AG). The subject was asked to walk at comfortable speed down the 10 m walkway. Data were collected for three successful trials by specialized technician. A set of 54 clinically relevant variables were selected a priori from kinematic and spatio-temporal parameters (Benedetti et al. 1998; Desloovere et al. 2006). The Gait Deviation Index, index of impaired walk, was calculated. (Schwartz and Rozumalski 2008). The GDI is based on the calculation of the distance between the patient’s data and the average from the gait lab reference dataset on 15 gait features of kinematics of the pelvis, hip, knee and ankle. This dataset included 1078 children with CP, and is matched on age with individuals of this study. The more impaired lower limb, having the lower GDI, was considered for the statistical analysis.

#### Clinical examination

Neuro-orthopaedic examination was standardized: joint range of motion, strength, spasticity and selective control motor of hip, knee and ankle’s muscles were described by 28 variables from literature review and clinical experience (Gage et al. 2009; Avers and Brown 2013; Viehweger et al. 2014). Physical examination was conducted by physiotherapists in accordance with a fully considered detailed protocol, which was writing prior in the Rehabilitation centre.

## Statistical analysis

The statistical analyses were performed with RStudio version 3.3.3. For the three-dimensional gait analysis and the physical examination, only the more affected lower limb was considered for the final analysis (Sangeux et al. 2013). The level of association between the kinematics, the spatio-temporal parameters, the physical examination measurements, the average daily step count and the levels of activity were tested with a Spearman’s rank correlation coefficient (r). A Spearman test uses a non-parametric approach and thus does not require the assumption of a normal distribution of observations. The correlations were poor if r < 0.20; fair if 0.21 < r < 0.40; moderate if 0.41 < r < 0.60; good if 0.61 < r < 0.80; very good 0.81 < r < 1.00 (Altman 1990).

A series of multiple linear regression analyses was made to correlate MVPA, SED and steps per day of children from gait analysis and clinical parameters, after Bonferroni’s corrections. The physical activity data were entered as the dependent variables and the clinical measurements and gait analysis data were used as the independent variables. After splitting the dataset into test set and training set (ratio 80/20), the backward elimination approach was used to select the optimal model with correlated variables. The coefficient of determination R-squared (R^2^), percentage of variance explained by the independent variables to determine a dependant variable, was calculated and adjusted. The standard error of the regression (S) was used to estimate the precision of the model, the threshold considered was S < 2.5 to produce a sufficiently narrow 95% interval. Predictive R-square (Predi-R^2^) was calculated, to tell how well a regression model determines responses for new observations. A pre-selection was made, because the number of participants was not enough to include all variables in the initial model. For MVPA, SED and steps per day, all independent variables that respect: p < 0.2 and r > 0.5 were included. The initial model evaluated the correlated value of 12 clinical measurements (hip extension, hip abduction evaluated with hip and knee flexed, hip abduction evaluated with hip and knee extended, hip external rotation, bilateral popliteal angle, ankle dorsiflexion evaluated with knee at 0°, femoral anteversion, tibio-femoral angle, Duncan Ely score and strength of hip abductors and ankle dorsi- and plantar flexors) and 9 spatio-temporal and kinematics parameters from gait analysis (step length, timing of toe-off, gait velocity, pelvic range of sagittal motion, hip flexion angle at terminal stance, hip rotation angle at toe-off, hip range of sagittal motion in stance, hip flexion velocity in swing, ankle maximum dorsiflexion in stance phase).

In subgroup analyses, children with a Global Motor Function Classification System II/III were grouped together to obtain consistent group sizes. Student tests were performed to compare MVPA, sedentary time and steps per day averages between children with a GMFCS I versus children with a GMFCS II/III.

A significance threshold of 0.05 was adopted for all statistical analyses.

## Results

Thirty patients were included in the final analysis. There is no missing data and no patients were excluded. There were 17 children with GMFCS I, 7 with GMFCS II and 6 with GMFCS III. There were 11 patients with hemiplegia and 19 with diplegia. For the 30 participants with seven full days of accelerometer data, the median daily step count was 5871 (range 1781–12551). Mean percent of time spent in MVPA was 14.6% over 10 hours (range 4.4%-30.8%), in other words 88 minutes per day (range 31–222 minutes per day). Mean percent of time spent in SED was 76.2% over 10 hours (range 52.1%-90.1%), in other words 457 minutes per day (range 313–541 minutes per day), more than 7 hours a day. Children with a GMFCS I practiced more daily physical activity (MVPA and steps per day) than children with a GMFCS II–III. Mean MVPA for children with a GMFCS I was 96.13 minutes (SD 35.55), whereas for children with a GMFCS II/III it was 74.02 minutes (SD 32.09). Mean SED for children with a GMFCS I was 449.83 (SD 50.94), mean SED for children with a GMFCS II/III was 469.75 minutes (SD 32.09). The median steps per day for children with a GMFCS I over 10 hours was 6385 (±2329) whereas for children with a GMFCS II/III was 5030 (± 3042).

Detailed information is presented in Table 1. Daily diary of patients showed that only one third of children have claimed that they have practiced sports or exercise. In this study, the sports or activities that the participants declared were: dance, gymnastics, cycling, swimming, horse riding, playing games, wheelchair basketball but the essential of the physical activity recorded is induced by locomotion and school playtime.

**Table 1.** Descriptive characteristics of the participants by Gross Motor Classification Scale (GMFCS)

Characteristics N = 30	Overall Mean (SD) N = 30	GMFCS I Mean (SD) N = 18	GMFCS II Mean (SD) N = 5	GMFCS III Mean (SD) N = 6	GMFCS II–III Mean (SD) N = 11
Age	12.1 (2.9)	12.5 (2.7)	12.4 (2.6)	10.7 (3.9)	11.5 (3.3)
Gender [N(%)]					
Male	15 (50)	11 (61)	2 (40)	2 (33)	4 (36)
Female	15 (50)	7 (39)	3 (60)	4 (77)	7 (64)
GDI	68.5 (11.3)	72.4 (11.6)	64.2 (3.6)	60.1 (9.6)	62.1 (7.4)
Walking speed	0.9 (0.2)	1.0 (0.2)	1.0 (0.2)	0.6 (0.1)	0.8 (0.3)
SED (%)	76.2 (8.2)	75.0 (8.5)	79.0 (8.7)	77.7 (7.3)	78.3 (7.6)
SED (min)	457.4 (49.0)	449.8 (57.9)	473.9 (52.1)	466.3 (43.6)	469.7 (45.3)
MVPA (%)	14.6 (5.9)	16.0 (6.0)	12.2 (6.3)	12.4 (5.1)	12.3 (5.3)
MVPA (min)	87.7 (35.4)	96.1 (35.6)	73.3 (37.7)	74.6 (30.3)	74.0 (32.1)
Steps per day	5871.4 (2654.5)	6385.5 (2329.3)	5660.6 (4415.2)	4504.7 (1476.1)	5030.1 (3041.6)

GDI: Gait Deviation Index; SED: sedentary time; MVPA: moderate to vigorous physical activity.

The mean GDI in total population was 68.5 (range 46.2–93.2). There was no correlation between the GDI and SED (r = −0.11, p = 0.55), MVPA (r = 0.24, p = 0.21) or steps per day (r = 0.33, p = 0.07).

None of the kinematic parameters were correlated with physical activity. Fair to moderate correlations were found between spatio-temporal parameters and physical activity. Timing of toe-off (r = −0.40, p < 0.03) correlated with MVPA. Walking-speed did not correlate with physical activity (r_MVPA_ = 0.13, r_SED_ = −0.06, r_Steps_ = 0.20, p > 0.05). Few clinical measurements of range of motion, strength and selective motor control were correlated with physical activity. Hip range of motion, strength and selective motor control were correlated with physical activity, such as hip flexor selective motor control (r = 0.69 with MVPA and r = 0.69 with steps per day), maximal hip flexion (r = 0.43), maximal hip rotation (r = 0.45), maximal hip abduction (r = 0.52) with MVPA only. The correlations founded are summarized in Tables 2 and 3.

**Table 2.** Correlation coefficients between gait analysis and time spent in MVPA, sedentary and steps per day

Parameters	Sedentary	MVPA	Steps per day
Gait pathology index			
Gait deviation index	−0.12	0.24	0.33
Time and distance Timing of TO	0.39*	−0.40*	−0.28

Significates codes: ‘*’ 0.05

**Table 3.** Correlation coefficients between clinical examination and time spent in MVPA, sedentary and steps per day

Parameters	Sedentary	MVPA	Steps per day
Hip			
Extension with knee extended Extension with knee flexed	0.44* – –	−0.48* – –	−0.46* – –
Flexion Internal rotation External rotation Abduction with hip and knee flexed Abduction with hip and knee extended Flexors Selective control motor	−0.46* – – -0.50* -0.42* -0.49* -0.70*	0.43* – – 0.45* 0.39* 0.52* 0.69*	– – -0.38* 0.45* – – – – 0.70 *

Significates codes: ‘*’ 0.05

The results of the multiple linear regression model were:

- For MVPA, the correlated variables were: hip extension, hip abduction evaluated with hip and knee extended, hip abduction evaluated with hip and knee flexed, hip external rotation and ankle dorsiflexion evaluated with knee at 0°. The linear regression model was stable (standard error = 0.565). 54% of variation was explained by the model (R^2^ adjusted = 0.54). Predictive R^2^ indicated that this model was unable to determine new observations (Predi-R^2^ = 8.9%).
- For SED, the correlated variables were: hip external rotation, ankle dorsiflexion evaluated with knee at 0°, timing of toe-off and ankle maximum dorsiflexion in stance. The linear regression model was stable (standard error = 0.58). 52% of variation was explained by the model (R^2^ adjusted = 0.52). Predictive R^2^ indicated that this model was unable to determine new observations (Predi-R^2^ = 9.2%).
- For steps per day, the correlated variables were: hip external rotation, femoral anteversion, timing of toe-off, hip angle at terminal stance, hip rotation angle at toe-off and hip flexion velocity in swing. The linear regression model was stable (standard error = 0.66). 43% of variation was explained by the model (R^2^ adjusted = 0.43). Predictive R^2^ indicated that this model was unable to determine new observations (Predi-R^2^ = 14.0%).

## Discussion

This present study explored the link between the gait analysis parameters, the clinical examination and the physical activity recorded in free-living situations for children with cerebral palsy. The aim of this cross sectional study was to examine the correlations between: three-dimensional gait analysis, clinical measurements and physical activity.

The physical activity of children with CP is well documented, they practice less physical activity than typically developing children ones (Bjornson et al. 2007; Maher et al. 2007; Zwier et al. 2010; Ryan et al. 2015). The results of this study confirmed this trend. For the 30 participants, the median daily step count was 5582, which was far from the typical 13460 steps per day taken by young people without impairment (Bjornson et al. 2007). Nowadays, the recommendation for optimal health is 12,000 steps/day (U.S. Department of Health and Human Services 2018). Among the studied population, 27 respected the time spent in MVPA fixed by the US federal guidelines for youth, more than 60 minutes a day. Concurrent validity studies utilizing heart rate monitoring, direct observation, indirect calorimetry, whole room calorimetry and doubly labelled water have demonstrated the Actigraph® to be a valid measure of physical activity in TD children and adolescent (Krishnaveni et al. 2009; Crouter et al. 2013; Romanzini et al. 2014; De Craemer et al. 2015; Johansson et al. 2016; Meltzer et al. 2016; Migueles et al. 2017), and in youth with CP (Capio et al. 2010; Clanchy et al. 2011; Gorter et al. 2012; O’Neil et al. 2014; Oftedal et al. 2014). Clanchy et al. showed that the Freedson’s algorithm is sufficiently sensitive (81,8%), specific (67,5%) and accurate (74,6%) for MVPA threshold in this population (Clanchy et al. 2011). However, Gaba et al. showed that, out of a population of 306 healthy children, differences in the level of MVPA, varied from 27 min/day (Puyau algorithm 2002) to 231 min/day (Freedson algorithm 2005) (Gába et al. 2016).

Regarding their daily diary, only one third of children have claimed that they have practiced sports or exercise. Personal barriers to explain the lack of exercises were related to physical abilities (lake of energy, fatigue, pain, fear of an injury, difficulty to learn required motor skill) or related to psychological factors (decreased motivation, feeling like an outsider, insecure or ashamed, cognitive impairment, not to be accepted by the peers) (Verschuren et al. 2012).

Children with GMFCS I practiced significantly more daily physical activity than GMFCS II–III (Van Wely et al. 2012; Bjornson et al. 2014; Balemans et al. 2015). Our results as well as the daily diary data collected confirm this.

In this study, if few spatio-temporal and clinical examination parameters correlated with intensity and quantity of physical activity, the links found were not enough to strongly correlate these parameters (R^2^ Є [0.43–0.54]; moderate R^2^ for each model). The correlated variables selected for the multiple regression analysis were based on previous paper on the impact of gait analysis and clinical examination parameters on gait impairments in children with CP (Desloovere et al. 2006; Gage et al. 2009).

Moderate correlations were found between the hip range of motion, strength and selectivity and MVPA (0.39 < r < 0.52). Kinematics and spatio-temporal parameters did not correlate with the physical activity. Furthermore, the GDI, a multivariate measure of gait abnormality, did not correlate with the number of steps per days, or with the time spent in sedentary activity or in MVPA.

Wilson et al. shown there was a correlation between the GDI and percentage spent in moderate intensity of physical activity (r = 0.47), the GDI and high physical activity (r = 0.51) and the GDI and the average steps per day (r = 0.58) (Wilson et al. 2015). Their results were not confirmed in this study. The methodological differences could explain that. First, inclusion criteria were not the same (GMFCS I–III included in our final analysis). Second, the tool used (Actigraph GT3X versus Step Watch Activity Monitor) was not worn during the same period (7 days vs. 2 days for Wilson et al.). Lastly, we considered the more impaired lower limb for the statistical analysis, whereas Wilson et al. used the mean GDI of both lower limbs. In our opinion, we considered that the more impaired lower limb may be the main barrier in practicing physical activity.

According to the results, it could be hypothesised that improving the gait quality may not systematically improve the level of physical activity. Reciprocally, abnormal quality of walking may not be a personal barrier to practice a physical activity.

Furthermore, the data recorded on gait abnormality in the laboratory, and the data collected by accelerometer in free-living situation should be considered as complementary.

Although this was an exploratory study, unable to determine a causal link between the quality of walking and the quantity/intensity of physical activity, this was a first approach to explore this relation. It appeared that the parameters of clinical examination and gait analysis were not sufficiently correlated to physical activity. Specific evaluation of physical activity appeared essential to complete the follow-up of children with CP. Furthermore, the study of physical activity in typical children showed that there were large disparities in the practice of physical activity, even though they were not affected by walking disturbances. It was important to see, however, whether it was the same in children with CP. Other studies could be carried out before-after treatment to study if there is a causal relation between an improvement in walking quality and physical activity.

### Study limitations

The analysis of more impaired lower limb was a potential limitation, because of the heterogeneity of population, participants with both unilateral and bilateral CP were included.

The validity of measures in sedentary activity with accelerometer is recently discussed, and more research is needed to validate this tool in children with CP (Innes and Darrah 2013). All included children were undergoing a clinically indicated three-dimensional gait analysis for future treatment (including single event multi-level surgery or botulinum neurotoxin treatment). It could be a bias avoiding generalization of the results. This study is an exploratory study where multiple parameters were observed in a specific population. This hypothesis-generating approach, could be biased, but it was chosen as the first step in order to understand the relationship between physical activity and clinical parameters.

## Conclusion

The correlation between kinematics, spatio-temporal parameters and clinical examination parameters are low to moderate.

Gait analysis and clinical examination could only partially reflect the overall level and quantity of daily physical activity. None of the tested models from three-dimensional gait analysis and clinical examination strongly determines physical activity. Regarding the weakness of the observed correlations, it is recommended to associate gait analysis and actimetry in the longitudinal follow-up of patients with CP. Futhermore, if the benefits of treatments such as Single Event Multi Level Surgery have already been demonstrated concerning gait, it would be interesting to test its effects on physical activity on children with CP.
